# Forecasting hand-foot-and-mouth disease cases using wavelet-based SARIMA–NNAR hybrid model

**DOI:** 10.1371/journal.pone.0246673

**Published:** 2021-02-05

**Authors:** Gongchao Yu, Huifen Feng, Shuang Feng, Jing Zhao, Jing Xu

**Affiliations:** Department of Gastroenterology, The Fifth Affiliated Hospital of Zhengzhou University, Zhengzhou, Henan, People’s Republic of China; South China University of Technology, CHINA

## Abstract

**Background:**

Hand-foot-and-mouth disease_(HFMD) is one of the most typical diseases in children that is associated with high morbidity. Reliable forecasting is crucial for prevention and control. Recently, hybrid models have become popular, and wavelet analysis has been widely performed. Better prediction accuracy may be achieved using wavelet-based hybrid models. Thus, our aim is to forecast number of HFMD cases with wavelet-based hybrid models.

**Materials and methods:**

We fitted a wavelet-based seasonal autoregressive integrated moving average (SARIMA)–neural network nonlinear autoregressive (NNAR) hybrid model with HFMD weekly cases from 2009 to 2016 in Zhengzhou, China. Additionally, a single SARIMA model, simplex NNAR model, and pure SARIMA–NNAR hybrid model were established for comparison and estimation.

**Results:**

The wavelet-based SARIMA–NNAR hybrid model demonstrates excellent performance whether in fitting or forecasting compared with other models. Its fitted and forecasting time series are similar to the actual observed time series.

**Conclusions:**

The wavelet-based SARIMA–NNAR hybrid model fitted in this study is suitable for forecasting the number of HFMD cases. Hence, it will facilitate the prevention and control of HFMD.

## Introduction

Hand-foot-and-mouth disease (HFMD) is an acute infectious disease caused by enterovirus, which is prevalent among young children [1]. Most cases are mild and self-limiting with symptoms of fever and herpes_(or rash)_on the hands, feet, and mouth [2]. However, few children may experience severe complications, such as meningitis, brainstem encephalitis, neurogenic pulmonary oedema, pulmonary haemorrhage, and circulatory failure [1, 2]. An effective treatment for HFMD does not exist [3]; therefore, prevention and control are particularly important. Although the EV71 vaccine has been introduced and the number of cases of EV71 and CA16 have decreased, other enteroviruses have increased gradually [1, 4]. The incidence of HFMD remained high [4–6]. Active early intervention is important. If accurate forecasting can be performed, then response can be provided in advance, thereby decreasing the incidence of HFMD and reducing the disease burden. Therefore, reliable forecasting is extremely important for the prevention and control of HFMD.

Scholars have used many types of models to forecast the incidence of HFMD. Among those models, the traditional autoregressive integrated moving average (ARIMA) model has been widely utilized [7–9]. Linearity is the necessary condition of its application. However, time series in the real world are often uncertain and complex [10], particularly the epidemic time series [11], and may contain both linear and nonlinear structures [12, 13]. The ability of the seasonal autoregressive integrated moving average (SARIMA) model to fit non-stationary time series is limited [14, 15]. A previous study [16] that compared the performances of the SARIMA model and Back-Propagation neural networks, demonstrated that the former was inferior to the latter. Some practical studies have demonstrated that the prediction effect of the SARIMA model was worse than that of hybrid models combined with neural networks [13, 17].

Artificial neural networks(ANNs), which are adaptive and nonlinear [18, 19], are appropriate for excavating nonlinear relationships in time series [18]. Owing to their powerful nonlinear mapping ability, it is assumed that they can achieve any desired accuracy [10]. Among them, the nonlinear autoregressive neural network(NARNN), a dynamic neural network, is suitable for time series forecasting [14, 15] Owing to its dynamic property and high fault tolerance performance [20]. Some scholars name it the neural network nonlinear autoregressive(NNAR) model. However, some scholars mentioned that the ANN model cannot extract linear patterns of data as well as nonlinear [21]. Using ANN model alone may not be the best solution for real-world time series [15].

In recent years, combined models have emerged to overcome the shortcomings of single models and improve the prediction accuracy [22, 23]. Typically, SARIMA models are combined with ANN models [14, 24, 25]. This combination has been applied in HFMD forecasting [26], wherein the SARIMA model fits linear relationships, whereas the ANN fits nonlinear relationships. Such a combination utilizes the unique strength of both models adequately and improves forecasting accuracy. However, some researchers [14, 21] argued that hybrid models do not necessarily outperform its constituents’ performances.

The models discussed above performed well in time series forecasting, but they are not absolutely perfect. Better forecasting models still need to be explored. Wavelet analysis has been used as a data preprocessing method and combined with other forecasting models in environmental science [27], hydrology [18] and financial time series [28]. It does not require the stationarity of time series, which is often the basic requirement of traditional methods [11]. Therefore, it is suitable for nonstationary and noisy signal processing [29]. In some studies [18, 30], wavelet analysis was performed to decompose original series into approximation component and detail components. The approximation component, which is similar to the original data but smoother, was used to construct the SARIMA model. Meanwhile, the detail components, which are high-frequency and may contain noise, were utilized to establish an ANN model. Subsequently, the forecast from the SARIMA model and ANN models were summed up to obtain the final forecasting results. The results of the studies indicated that the wavelet-based combined model was superior to single models [30]. However, this type of model has not been used to forecast HFMD cases hitherto.

Hence, we propose to fit a wavelet-based SARIMA–NNAR hybrid model to forecast the number of HFMD cases. We are expected that this model is suitable for forecasting HFMD cases and facilitate the prevention and control of HFMD.

## Materials and methods

### Data collection and processing

The weekly number of HFMD cases was obtained from the Zhengzhou Center for Disease Control and Prevention, China. The information contained no missing data and no personal information was recorded. Therefore, ethics approval and consent are not necessary. We segmented the data into a training set and a validation set. In the training set, the weekly number of HFMD cases from 2009 to 2015 were used to fit models. While in the validation set, the weekly number of HFMD cases in 2016 were used to estimate the performance of the models. We plotted the time series and used the “stl” function in the “stats” package of “R” software to decompose the time series to investigate its trend, seasonality and error.

### Establishing SARIMA model

Autoregressive integrated moving average(ARIMA) model is one of the mostly used models to forecast the number of cases of infectious diseases. If a seasonal component is included, then the model can be known as seasonal autoregressive integrated moving average(SARIMA) model. Generally, it is denoted as SARIMA(p, d, q)(P, D, Q)s, where p is the order of the autoregressive(AR) model, d the number of difference, q the order of the moving average(MA) model, P the order of the AR seasonal model, D the number of seasonal difference, Q the order of the MA seasonal model, and s the length of the seasonal period. The formula of SARIMA(p, d, q)(P, D, Q)s is as follows [30].

$$\phi (B)\Phi (B^{\text{s}}){\left(1-B\right)}^{\text{d}}{\left(1-B\right)}^{D\text{s}}{\text{y}}_{\text{t}}=\theta (B)\Theta (B^{\text{s}})\varepsilon_{\text{t}}$$ (1)

Here, B signifies the backward shift operator, and ε_t_ denotes the residual. ϕ(B) = 1-ϕ_1_B-…-ϕ_p_B^p^; θ(B) = 1-θ_1_B-…θ_q_B^q^; Ф(B^s^) = 1-Ф_1_B^s^-…-Ф_p_B^Ps^; Θ(B^s^) = 1-Θ_1_B^s^-…-Θ_Q_B^Qs^

Owing to the obvious seasonality of the HFMD cases and the one-year period investigated, a SARIMA model was constructed, and s was set to 52 weeks.

Firstly, stationarity is required to fit a SARIMA model. The Augmented Dickey–Fuller (ADF) unit root test is frequently used to test the stationarity. Differencing and seasonal differencing are often used to transform the nonstationary series into a stationary series. Second, the order of the model is selected based on the autocorrelation function_(ACF) and partial autocorrelation function_(PACF). Subsequently, an optimal model is selected based on the Akaike information criterion (AIC) and Bayesian information criterion [31, 32] and the model parameters are estimated. Finally, the residuals are examined with the ACF, PACF and Box–Ljung test. The residuals are supposed to be white noise and have no autocorrelation.

We used the “auto.arima” function in the “forecast” package of “R” software to fit models with different values of p, d, q, P, D, and Q. Subsequently, the best model was selected by minimizing AICc, which is the corrective AIC. Given the same value of d and D, the minimum AICc corresponds to the best model.

### Building NNAR model

Artificial neural networks are based on mathematical models of the brain. The basic structure includes an input layer, hidden layers, and an output layer. An example of structure of ANN model was shown in S1 Fig. (This figure was obtained from https://otexts.com/fpp2/nnetar.html).In this study, we used an neural network nonlinear autoregressive(NNAR) model [33], which is a feed-forward neural network with a single hidden layer, and lagged values of the time series as inputs. It is denoted as the NNAR(p,P,k)_m_ model, where p is the number of inputs lags, P the seasonal lags, k the number of nodes in the hidden layer and m the length of the seasonal period. The formula of the NNAR(p,P,k)_m_ model is as follows [33].

$${\text{y}}_{\text{t}}=\text{f}({\text{y}}_{\text{t}-1},{\text{y}}_{\text{t}-2},\ldots ,{\text{y}}_{\text{t}-\text{p}},{\text{y}}_{\text{t}-\text{m}},{\text{y}}_{\text{t}-2\text{m}},\ldots ,{\text{y}}_{\text{t}-\text{Pm}})+\varepsilon_{\text{t}}$$ (2)

Here, f represents the neural network with k hidden nodes in a single layer, and ε_t_ is the residual series.

The “nnetar” function in the “forecast” package of R software can automatically obtain the optimal parameter p, P, and k. For seasonal time series, the default value is P = 1, and p is selected from the optimal linear model fitted to the seasonally adjusted data. If k is not specified, then it is set to k = (p + P + 1)/2 (rounded to the nearest integer) [33].

### Constructing SARIMA–NNAR combined model

In the first place, a SARIMA model was fitted. Subsequently, its residual series were inputted to the NNAR model. The nonlinear relationships that the residuals may contain can be mined adequately by neural networks. The final combined forecasting values of the time series were the sum of predictions from the SARIMA model and the adjusted residuals from NNAR model. The structure of the SARIMA–NNAR combined model is shown in S2 Fig.

### Formulating wavelet-based SARIMA–NNAR hybrid model

We used discrete wavelet transformatiom, which is often used in time series decomposition [30]. Different wavelets exist, such as Daubechies, Coiflets, and Symlets. Through literature review [18, 30], we selected a Daubechies wavelet, which is denoted as “db2” in MATLAB, and one or two decomposition levels. The “db2” wavelet was used to decompose the original data into an approximation component and detail components in different levels (one or two). The approximation component is low-frequency and similar to the original data but smoother. The detail components are high-frequency which usually contain noise. Afterwards, a SARIMA model was fitted to the approximation component, whileas a NNAR model was fitted to the detail components. The final results were computed by summing the results from the SARIMA and NNAR models. Wavelet decomposition and reconstruction were performed in MATLAB software(Version R2014a). The structure of the wavelet-based SARIMA–NNAR hybrid model is shown in S3 Fig.

### Performance evaluation of four models

The models fitted with the training set were used to forecast forward 52 weeks. The number of cases in every week were forecasted based on previous value. Three indices were computed to measure the accuracy of fitness and forecasting for the four models:the root mean square error(RMSE), mean absolute error (MAE) and mean absolute percentage error (MAPE). These indices are expressed as follows:

$$\text{RMSE}:\sqrt{\frac{1}{\text{n}}\sum_{\text{t}=1}^{\text{n}}{\left({\text{y}}_{\text{t}}-\overset{\wedge }{{\text{y}}_{\text{t}}}\right)}^{2}}$$ (3)

$$\text{MAE}:\frac{1}{\text{n}}\sum_{\text{t}=1}^{\text{n}}\left|{\text{y}}_{\text{t}}-\overset{\wedge }{{\text{y}}_{\text{t}}}\right|$$ (4)

$$\text{MAPE}:\frac{1}{\text{n}}\sum_{\text{t}=1}^{\text{n}}\frac{\left|{\text{y}}_{\text{t}}-\overset{\wedge }{{\text{y}}_{\text{t}}}\right|}{{\text{y}}_{\text{t}}}$$ (5)

Here, y_t_ represent the observed time series at time t and $\overset{\wedge }{{\text{y}}_{\text{t}}}$ the fitted or forecast time series.

## Results

### General information

**Fig 1:**
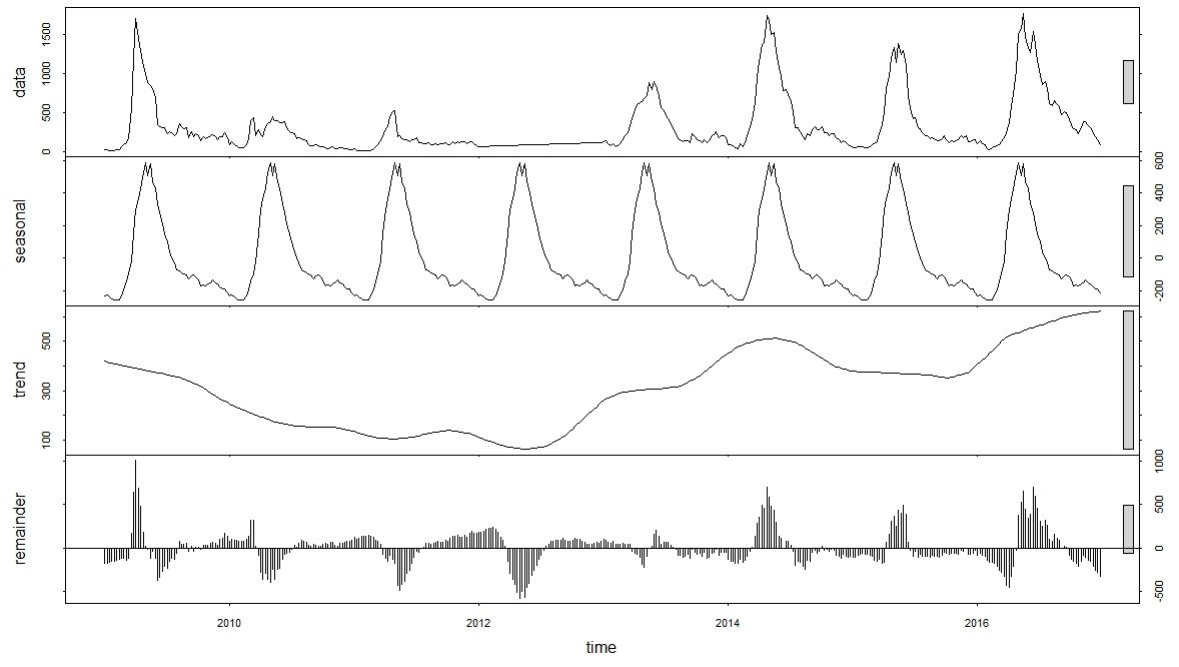
Time series plot of weekly HFMD cases from 2009 to 2016 in Zhengzhou, China.

A total of 128,682 cases have been reported in Zhengzhou, China from 2009 to 2016. The peaks of cases often occurred between May and July. The time series plot of the weekly HFMD cases, as depicted in Fig 1, shows clear seasonality based on the results of “stl” decomposition. A one-year period of HFMD prevalence was observed.

### Best-performing SARIMA model

**Fig 2:**
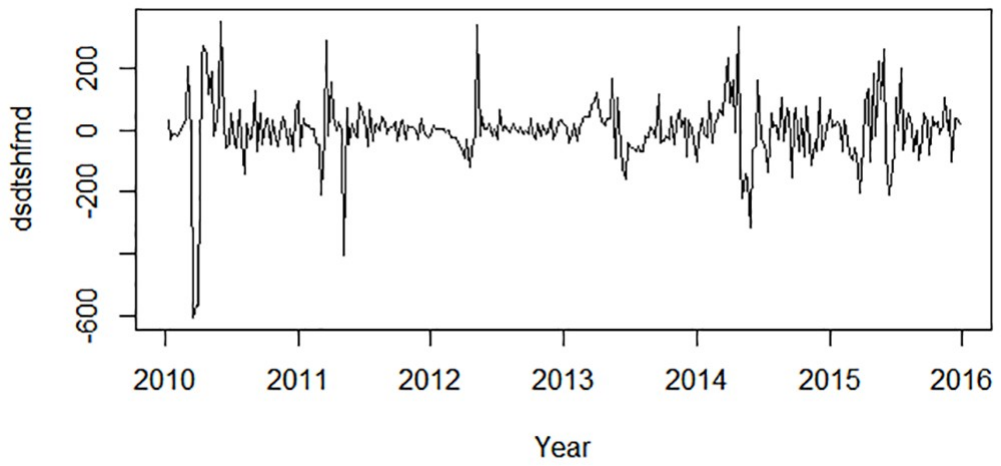
Regular differenced and seasonal differenced time series plot.

**Fig 3:**
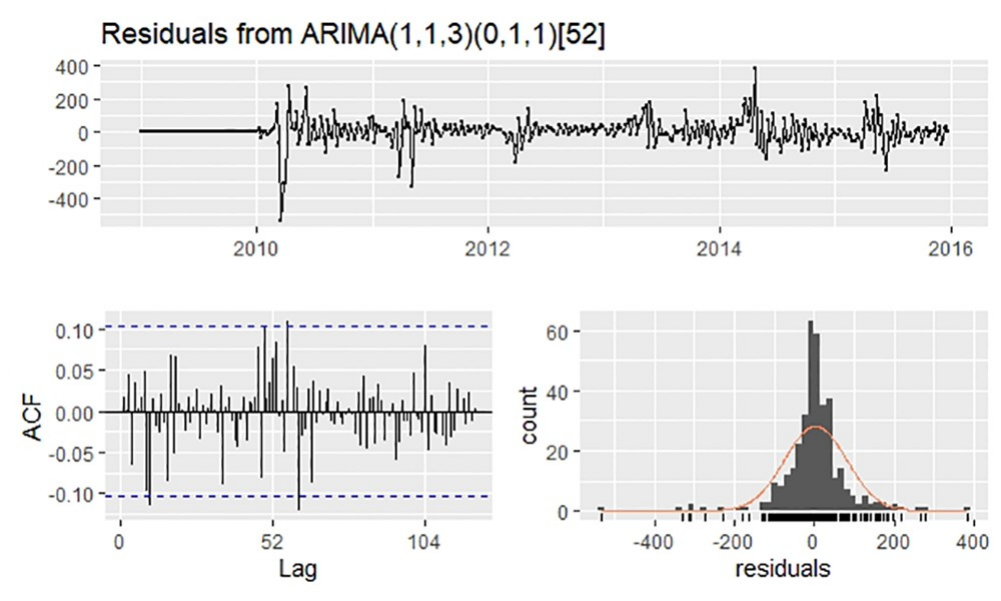
Residuals plot, corresponding ACF plot, and histogram from ARIMA(1,1,3)(0,1,1)52.

The ADF test implied that the time series was nonstationary (P = 0.4451). After one difference and one seasonal difference, the time series became stationary (ADF test P = 0.04478) (Fig 2). Using the “auto.arima” function with d = 1 and D = 1, the optimal model was selected as SARIMA (1,1,3)(0,1,1)_52_ with the lowest AICc of 3668.13. The residuals plot, the corresponding ACF plot, and a histogram are shown in Fig 3. The Ljung–Box test of the residuals, whose P value is 0.6359, demonstrated no autocorrelation in the residuals.

### Best-performing NNAR model

**Fig 4:**
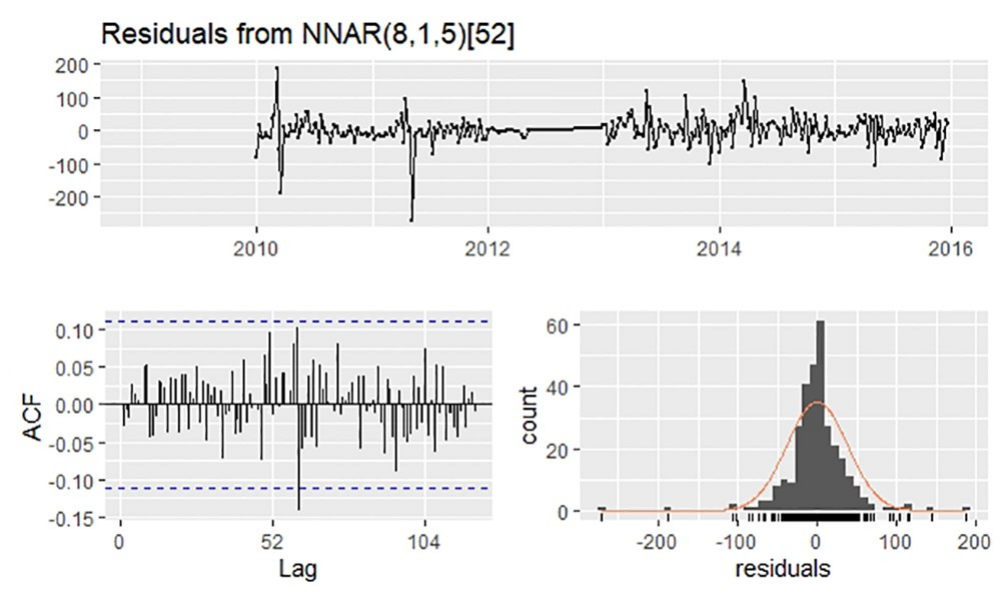
Residuals plot, corresponding ACF plot, and histogram from NNAR(8,1,5)52.

Owing to seasonality, P was set to 1. In addition to the automatic selection of (6,1,4)_52_ by the”nnetar” function, we tested different p values from 1 to 10 (Table 1). Considering three indices of performance both in training and validation sets, we finally selected NNAR(8,1,5)_52_ as the optimal model. The residuals are shown in Fig 4. The Ljung–Box test, whose P value was 0.5917, demonstrated no autocorrelation in the residuals.

**Table 1:** Accuracy of 10 candidate NNAR models in training and validation sets.

NNAR model	Training set			Validation set		
	RMSE	MAE	MAPE	RMSE	MAE	MAPE
(1,1,2)52	74.17	42.83	21.33	471.67	295.87	57.88
(2,1,2)52	64.66	38.75	22.91	515.23	355.63	68.88
(3,1,2)52	62.15	35.95	23.07	455.17	315.41	62.93
(4,1,3)52	53.84	32.13	21.46	394.90	274.97	64.58
(5,1,4)52	46.57	28.85	21.68	349.08	243.48	57.56
(6,1,4)52	45.94	28.36	20.65	359.88	262.90	62.48
(7,1,4)52	45.71	28.30	21.91	332.62	231.62	54.41
(8,1,5)52	37.90	23.77	19.53	309.44	211.96	57.48
(9,1,6)52	34.15	21.77	19.28	323.15	215.22	52.52
(10,1,6)52	33.58	20.56	19.67	344.24	230.52	55.00

#### Best-performing SARIMA–NNAR model

**Fig 5:**
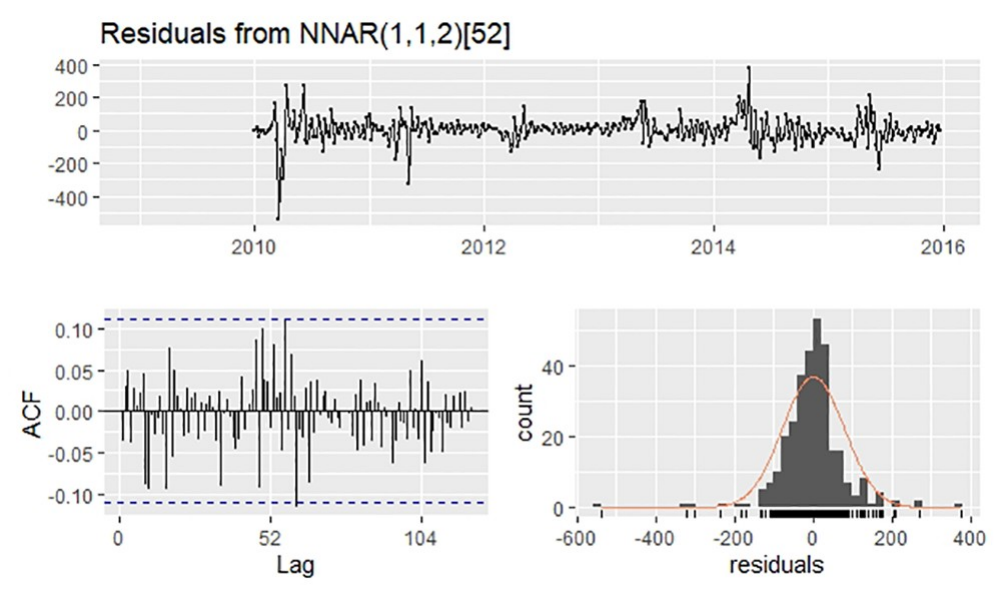
Residuals plot, corresponding ACF plot, and histogram from NNAR(1,1,2)52.

The SARIMA model was fitted as explained previously. The optimal NNAR model that employed the residual series generated from the SARIMA model was selected as NNAR(1,1,2)_52_. The residuals are shown in Fig 5. The Ljung–Box test, whose P value was 0.5199, demonstrated no autocorrelation in the residuals.

### Best-performing wavelet-based hybrid SARIMA–NNAR model

The “db2” wavelet decomposed the original data into an approximate component (cA) and detail components (cD) in one level or two levels (Table 2). The performances of the wavelet-based hybrid model using different decomposition levels are shown in Table 3. Based on the training set, two level of decomposition performed better, whereas based on the validation set, one level of decomposition was superior. In view that the purpose of model is forecasting, we regarded one level as the better decomposition level.

**Table 2:** Models for approximate and detail components and P value of Ljung-Box test for residuals.

Level of decomposition	SARIMA for cA	P value(cA)	NNAR for cD	P value(cD)
One	(4,1,0)(1,1,0)52	0.1476	(16,1,9)52	0.0546
Two	(4,1,1)(1,1,1)52	0.1198	(9,1,6)52	0.5681

cA: approximate component; cD: detail component. P value(cA): P value of Box. Test of residuals from SARIMA for cA. P value(cD): P value of Box. Test of residuals from NNAR for cD.

**Table 3:** Accuracy of optimal models with one or two wavelet decomposition levels.

Level of decomposition	Training set			Validation set		
	RMSE	MAE	MAPE	RMSE	MAE	MAPE
One	71.29	37.45	21.81	296.18	227.25	61.32
Two	56.67	28.46	19.12	314.34	240.74	61.45

### Accuracy comparison among four types of models

The performances of four types of models in the training and validation sets are shown in Table 4. In the training set, the RMSE, MAE, and MAPE of the single NNAR model were the lowest, followed by those of the wavelet-based hybrid model. As for the validation set, the values of the indices of the wavelet-based hybrid model were the lowest in most situations, except for MAE and MAPE of the single NNAR model.

**Table 4:** Accuracy of training set and 52 weeks forecasting in validation set.

	Training set			52 weeks		
	RMSE	MAE	MAPE	RMSE	MAE	MAPE
SARIMA	77.38	46.27	32.96	307.02	238.45	64.01
NNAR	37.90	23.77	19.53	309.44	211.96	57.48
SARIMA–NNAR	78.60	51.03	36.81	304.33	236.84	65.58
Wavelet hybrid	71.29	37.45	21.81	296.18	227.25	61.32

SARIMA–NNAR: regular SARIMA–NNAR hybrid model; Wavelet hybrid: wavelet-based SARIMA–NNAR hybrid model.

**Fig 6:**
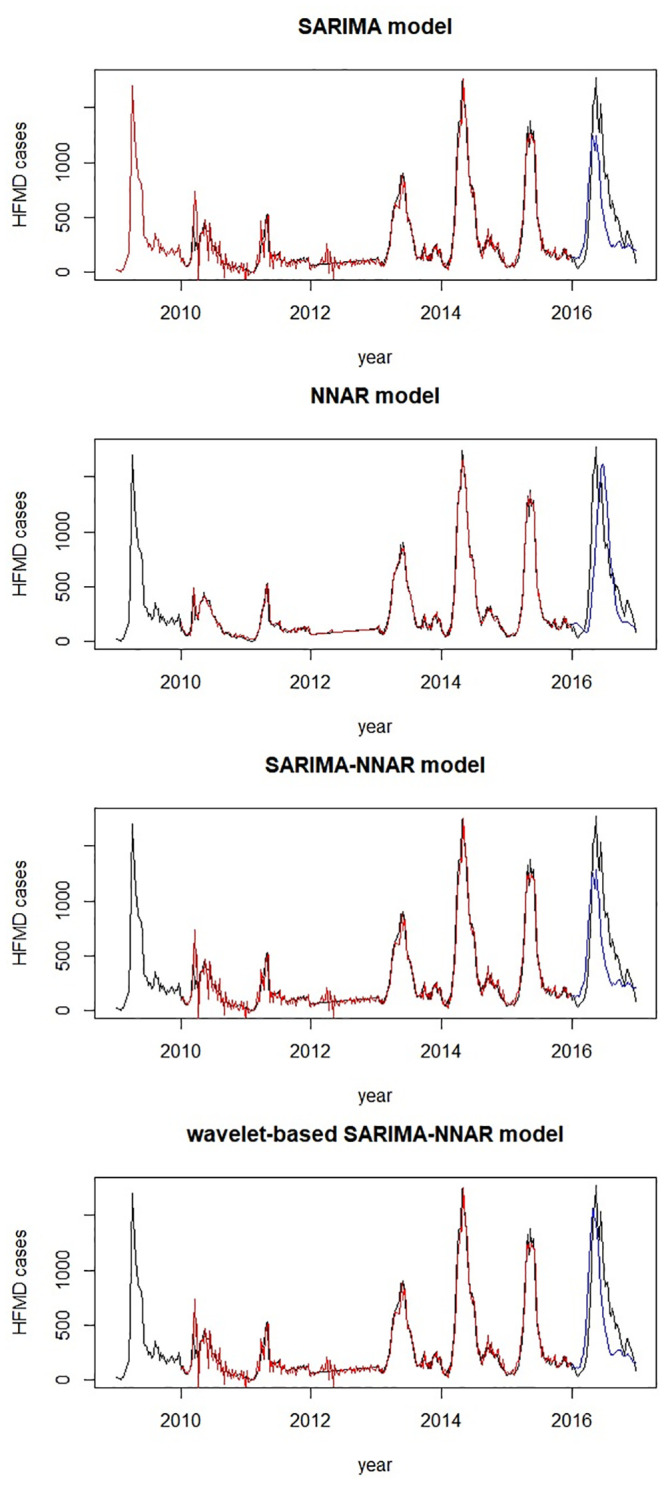
Fitted and 52 weeks forecasting time series plot of four models. Black line: original time series; red line: fitted time series in training set; blue line: forecasting time series in validation set.

The fitted and forecasting time series plot of four models are shown in Fig 6. In terms of the training set, all models fitted well, while the fitted time series by NNAR model was especially approximate to original data. As far as validation set is concerned, the peak of the wave forecasted by the single SARIMA model and the regular SARIMA-NNAR model were lower than that of the actual observed data, whereas that forecasted by the NNAR model deviated slightly from that of the real time series. It appeared that the wavelet-based hybrid model may performed better in the validation set compared with other models.

## Discussion

In this study, a wavelet-based SARIMA–NNAR hybrid model was fitted to forecast the number of HFMD cases with data from 2009 to 2016 of Zhengzhou, China. To estimate its performance, we compared it with the single SARIMA model, simplex NNAR model, and pure SARIMA–NNAR hybrid model. We discovered that the wavelet-based hybrid model demonstrated excellent performances whether in fitting or forecasting compared with other models. Its fitted and forecasting time series were approximate to the actual observed time series. This wavelet-based SARIMA–NNAR hybrid model is suitable for forecasting the number of HFMD cases and will facilitate the prevention and control of HFMD.

Wavelet analysis is a new and effective method for analyzing nonstationary and noisy signals. It does not require the stationarity of time series. As a powerful data preprocessing method, wavelet analysis has been combined with forecasting models to perform forecasting in certain areas. It is capable of decomposing original data into approximation and detail components in different levels. By allowing different components to be forecasted using different models and summing the results, this wavelet-based SARIMA–NNAR hybrid model enables the accuracy of forecasting to be improved.

However, some limitations exist in this study. First, the original data were collected from Zhengzhou Center for Disease Prevention and Control, which may have the possibility of false reporting and omissive reporting. The quality of data may affect the construction process and performance of model to some degree. Additionally, different types of wavelets and different levels of wavelet decompositions exist; moreover, different detail components can be modeled respectively. We selected only the”db2” wavelet and one or two decomposition levels. With two levels of decomposition, we obtained two detail components but just synthesized them to one total detail component through wavelet reconstruction. We did not investigate different wavelets, more decomposition levels, and different methods to manage the detail components. More trials might yield better results. Furthermore, this hybrid model should be updated timely to preserve its accuracy with new data. Finally, the influencing factors of HFMD are complex and various factors should be considered for its prediction, such as climate [34] and transmission dynamics [35].

## Conclusions

In this study, a wavelet-based SARIMA-NNAR hybrid model was fitted to forecast the number of HFMD cases with weekly data from 2009 to 2016 of Zhengzhou, China. The wavelet-based hybrid model had an excellent performance whether in fitting or forecasting compared with other models. The wavelet-based SARIMA–NNAR hybrid model is suitable for forecasting the number of HFMD cases, and will facilitate the prevention and control of HFMD.

## Supporting information

S1 Fig Example of structure of ANN model. (TIF)

S2 Fig Structure of the SARIMA–NNAR combined model. (TIF)

S3 Fig Structure of the wavelet-based SARIMA–NNAR hybrid model. (TIF)
