# Supplementary figures and images for: Forecasting hand-foot-and-mouth disease cases using wavelet-based SARIMA–NNAR hybrid model
Source: PLoS One. 2021 Feb 5;16(2):e0246673. doi: 10.1371/journal.pone.0246673 (PMC7864434; doi:10.1371/journal.pone.0246673)

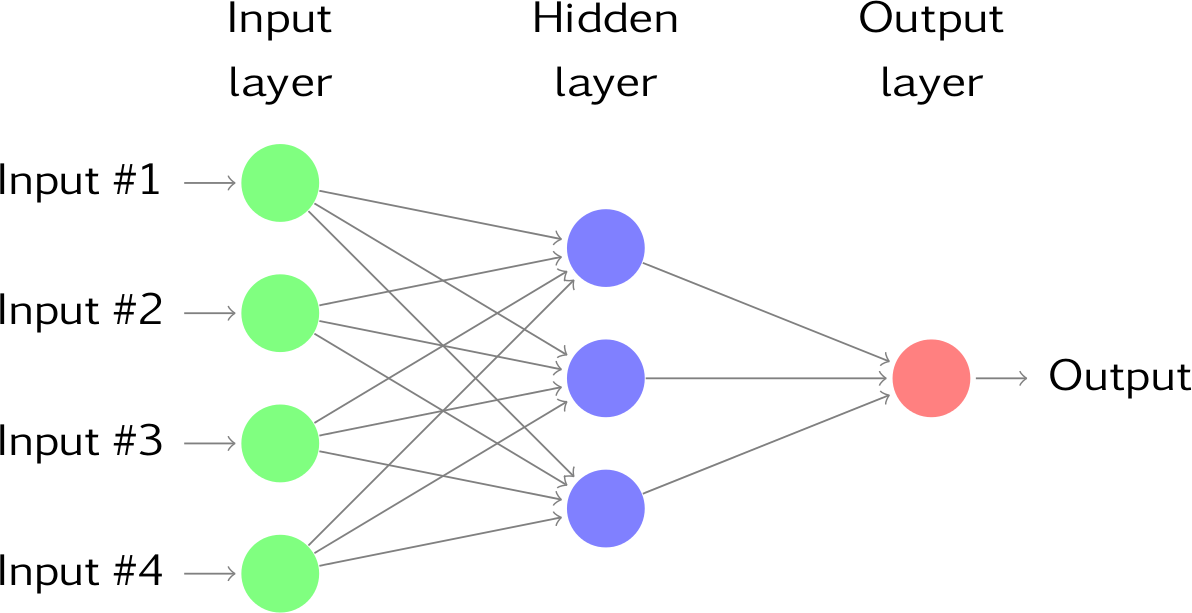

Supplement: S1 Fig — (TIF) [file pone.0246673.s001.tif]

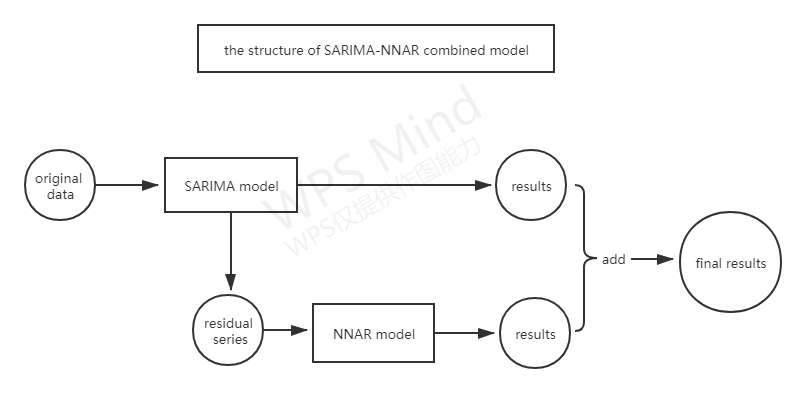

Supplement: S2 Fig — (TIF) [file pone.0246673.s002.tif]

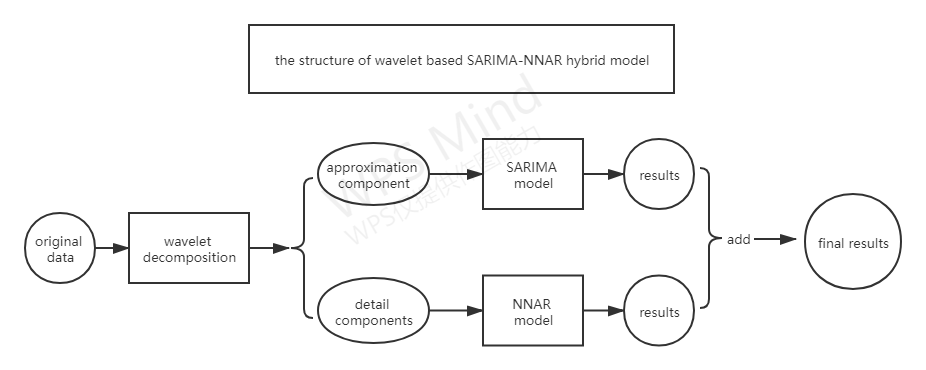

Supplement: S3 Fig — (TIF) [file pone.0246673.s003.tif]
